# A novel variant in *PAX6* as the cause of aniridia in a Chinese family

**DOI:** 10.1186/s12886-021-01848-z

**Published:** 2021-05-20

**Authors:** X Jin, W Liu, LH Qv, WQ X, HB Huang

**Affiliations:** 1 Department of Ophthalmology, Chinese PLA General Hospital, 100853 Beijing, China; 2 Department of Ophthalmology, Hainan Hospital of Chinese PLA General Hospital, 572000 Sanya, Hainan Province China; 3 Department of Ophthalmology, the 74th Army Group Hospital, 510318 Guangzhou, China; 4 The Second School of Clinical Medicine, Southern Medical University, Guangzhou, 510515 China

**Keywords:** Aniridia, Autosomal dominant inheritance, *PAX6* gene, Mutation

## Abstract

**Background:**

Aniridia is a kind of congenital human pan-ocular anomaly, which is related to *PAX6* commonly.

**Methods:**

The ophthalmic examinations including visual acuity, slit lamp and fundoscopy examination were performed in a Chinese aniridia pedigree. The targeted next-generation sequencing of aniridia genes was used to identify the causative mutation.

**Results:**

A novel heterozygous *PAX6* nonsense mutation c.619A > T (p.K207*) was identified in the Chinese autosomal dominant family with aniridia. Phenotype related to the novel mutation included nystagmus, keratopathy, absence of iris, cataract and foveal hypoplasia.

**Conclusions:**

The novel nonsense variation in *PAX6* was the cause of aniridia in this family, which expanded the spectrum of the *PAX6* mutation.

## Background

Aniridia was a kind of rare congenital human ocular anomaly, it could manifest either as ocular abnormalities of iris hypoplasia, foveal hypoplasia, cataract, glaucoma, corneal dystrophy, as well as abnormalities of the optic nerve, or as syndromes of the WAGR syndrome (MIM#194,072) and the Gillespie syndrome (MIM#206,700) [1, 2]. The incidence of aniridia was between 1 and 50,000 and 96,000 births [3]. According to the difference of pathogenic genes, aniridia could be divided into three types, aniridia-1 (AN1, MIM #106,210), aniridia-2 (AN2, MIM #617,141) and aniridia-3 (AN3, MIM #617,142). AN1 was caused by heterozygous mutation in the *PAX6* gene, AN2 was caused by heterozygous mutation in a *PAX6* cis-regulatory element that resides in an intron of the adjacent *ELP4* gene, and AN3 was caused by heterozygous mutation in the *TRIM44* gene [4, 5]. Some of the isolated aniridia was demonstrated due to mutations in *FOXC1* or *PITX2* genes. Mutations in these two genes were more commonly associated with juvenile onset glaucoma and anterior segment dysgenesis presenting with syndromic features of rare cardiac anomalies for *FOXC1* and hypodontia and umbilical anomalies for *PITX2* [6–8].

About one-third of the cases carry de novo variants [9]. The classic AN1 associated with *PAX6* haploinsufficiency presented iris hypoplasia and foveal hypoplasia, while heterozygous missense mutations in *PAX6* would lead to other ocular diseases including anterior segment dysgenesis and optic nerve malformations. *PAX6* (OMIM 607,108), paired box gene 6, was a member of the paired box gene family which encodes a transcriptional regulator involved in oculogenesis and other developmental processes [10]. This transcription factor had shown functional conservation in developmental pathways. *PAX6* variant had been identified associated with aniridia and other ocular development abnormalities previously, while olfactory abnormalities and brain structure alterations in line with expression of Pax6 had also been documented recently [11]. *PAX6* played an important role in the process of development regulation which broadly expressed and was controlled by a number of long-range control elements and homozygous mutation led to more severe phenotype [11].

In the present study, we identified a novel heterozygous *PAX6* nonsense mutation c.619A > T (p.K207*) in a Chinese autosomal dominant family with aniridia. We used targeted next-generation sequencing (NGS) to screen all the genes related to iris diseases including aniridia and identified the causative mutations.

## Methods

The Institutional Review Board (IRB) of Hainan hospital of Chinese PLA General Hospital (Hainan Province, China) approved the present study. All participating family members provided an informed written consent and were endorsed by their respective IRB. The whole procedure of the present study adhered to the tenets of the Declaration of Helsinki.

**Fig. 1:**
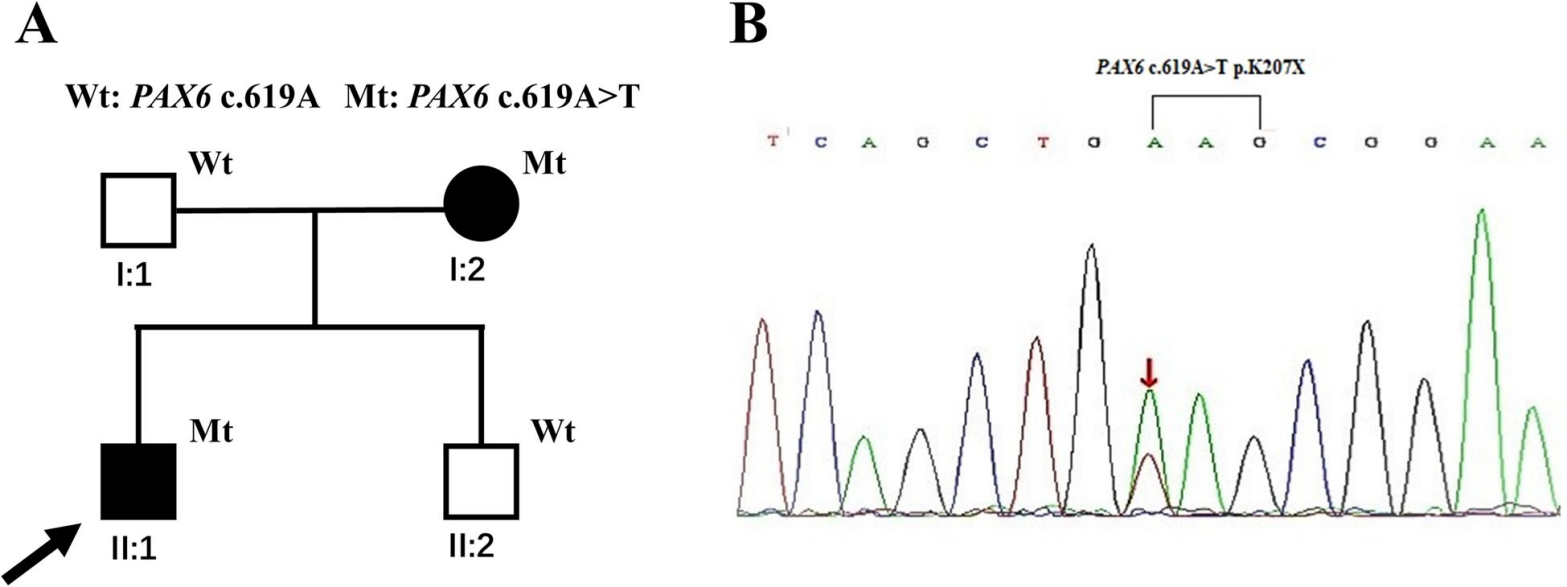
Identification of the heterozygous mutation c.619A > T in *PAX6* in a Chinese family with aniridia. **a** Pedigree of the family. Squares indicated males and circles indicated females. Empty symbols and filled symbols represented the normal and affected individuals, respectively. Wt: wild-type and Mt: mutation. **b** Sequence chromatograms showing the *PAX6* c.619A > T mutation identified in this study. The arrows indicated the site of the mutation

A small pedigree with aniridia from Hainan province, China, was recruited for the present study. This family included two affected and two unaffected members, which was analyzed and followed up clinically at Hainan Hospital of Chinese PLA General Hospital (Fig. 1a). Comprehensive ophthalmological examinations, including best correct visual acuity (BCVA), applanation tonometry, dilated funduscopy, anterior segment and fundus photography, ultrasound biomicroscopy (UBM) examination, gonioscopy and optical coherence tomography (OCT) of anterior segment and macular were performed on the affected individuals and the unaffected family members. Genomic DNA was collected from peripheral blood lymphocytes of the pedigree members and normal controls using a QIAamp DNA Blood Midi Kit (Qiagen, Hilden, Germany).

### Targeted genes enrichment and sequencing

Target enrichment panel of specific hereditary eye based on the next generation sequencing was used to collect the protein-coding region of 371 targeted genes (designed by MyGenostics, Baltimore, MD), which included 37 genes associated with iris diseases. (*PAX6, ELP4, FOXD3, PITX3, FOXE3, PITX2, ADAMTS10, FBN1, LTBP2, ADAMTS17, MTHFR, TYR, MITF, PAX3, SNAI2, SOX10, RBP4, CHD7, TMEM67, RPGRIP1L, CC2D2A, YAP1, MAF, C12orf57, FOXC1, B3GLCT, NF1, GPR143, OCA2, TYRP1, CRYGC, ADAMTSL4, SALL2, IGBP1, CYP1B1, MYOC, SALL1*). The genes related to aniridia, WAGR syndrome, Axenfeld-Rieger syndrom and other diseases involved iris abnormity were covered. The process of specific high throughput sequencing was in conformity to some published articles [12, 13].

### Bioinformatics analysis

After HiSeq 2000 sequencing, Solexa QA package and the Cutadapt program were used to filter out low quality reads and adaptor sequences and high-quality reads from raw reads were retrieved. Then the clean read sequences were aligned to the human reference genome (hg19) using SOA Paligner program. Subsequently, the single nucleotide polymorphism (SNPs) and the insertions or deletions (InDels) were identified using the SOAPsnp program and GATK program separately. The identified SNPs and InDels were annotated using ANNOVAR program (http://122.228.158.106/exomeassistant) and viewed using MagicViewer. Finally, nonsynonymous variants were evaluated by four algorithms, Ployphen, SIFT, PANTHER and Pmut, as described previously to determine pathogenicity.

### Mutation verification

After high throughput sequencing, the detected variations were validated by Sanger sequencing in the Chinese family. Primer6.0 was used to design the PCR primer sets and the PCR products were sequenced using a Bigdye terminator v3.1 cycle sequencing kit (ABI, Foster City, CA, USA) and analyzed on an ABI 3730XL Genetic Analyzer. The primers used in this study are listed in Table 1.

**Table 1:** Primers used for potential pathogenic mutations amplification

Mutation	Gene	Exon	Forward primer (5'-3' )	Reverse primer (5'-3' )
c.619A > T	*PAX6*	8	TCAGACATTTAGTCTTTGAATTACTGG	GAGTTTAAGACTACACCAGGCCC

## Results

### Clinical findings

**Fig. 2:**
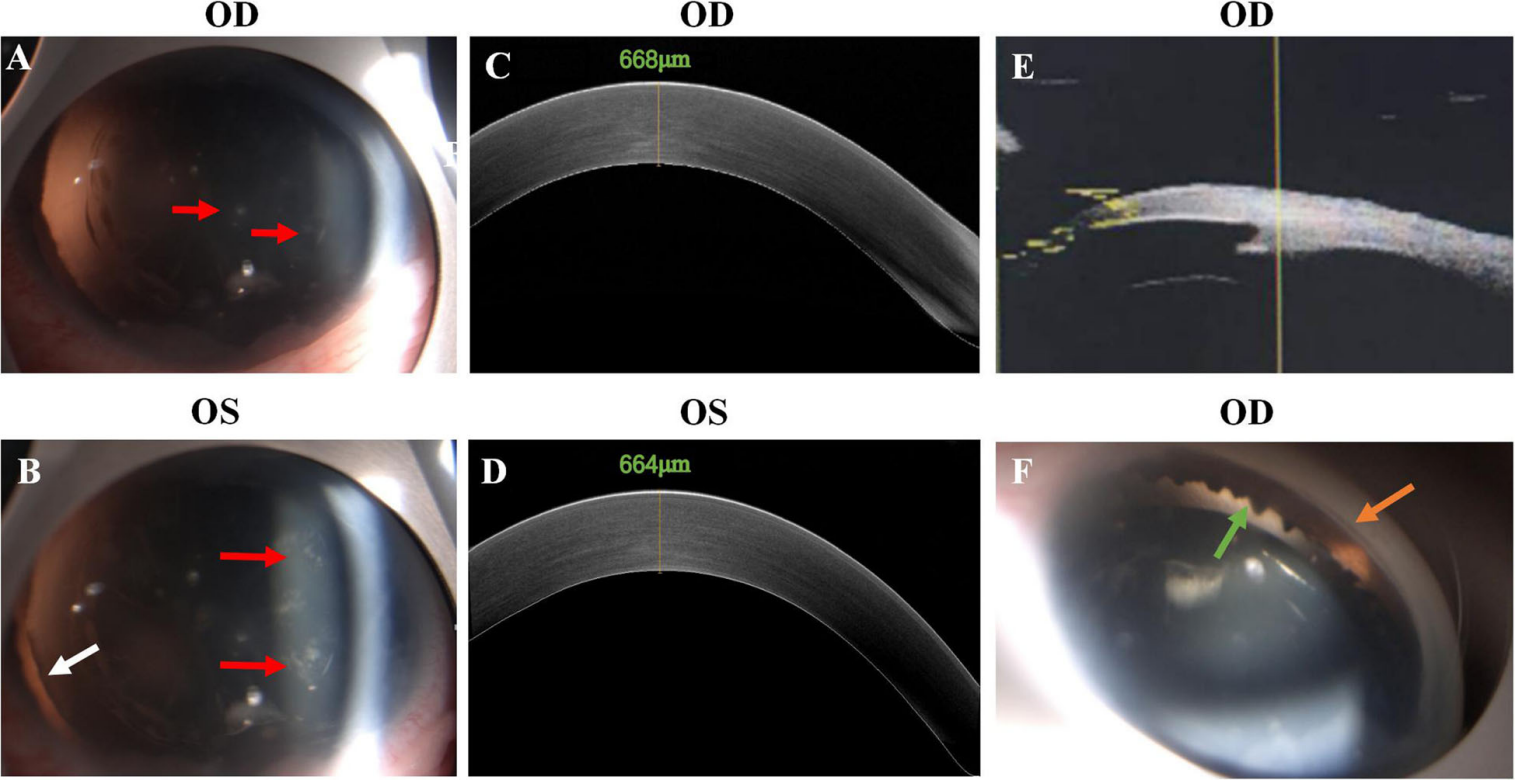
Anterior segment features of the proband in the family with aniridia. **a** and **b** Slit-lamp photographs of proband. The white arrow indicated the equator of lens and the red arrows indicated discrete posterior subcapsular cataract. **c** and **d** Cornea OCT examination of proband. **e** Ultrasound biomicroscopy examination of proband’s right eye. (F) Anterior gonioscopy photographs. The green arrow indicated ciliary process and the orange arrow showed the structure of anterior chamber angle. OD stand for right eye, OS for left eye

**Fig. 3:**
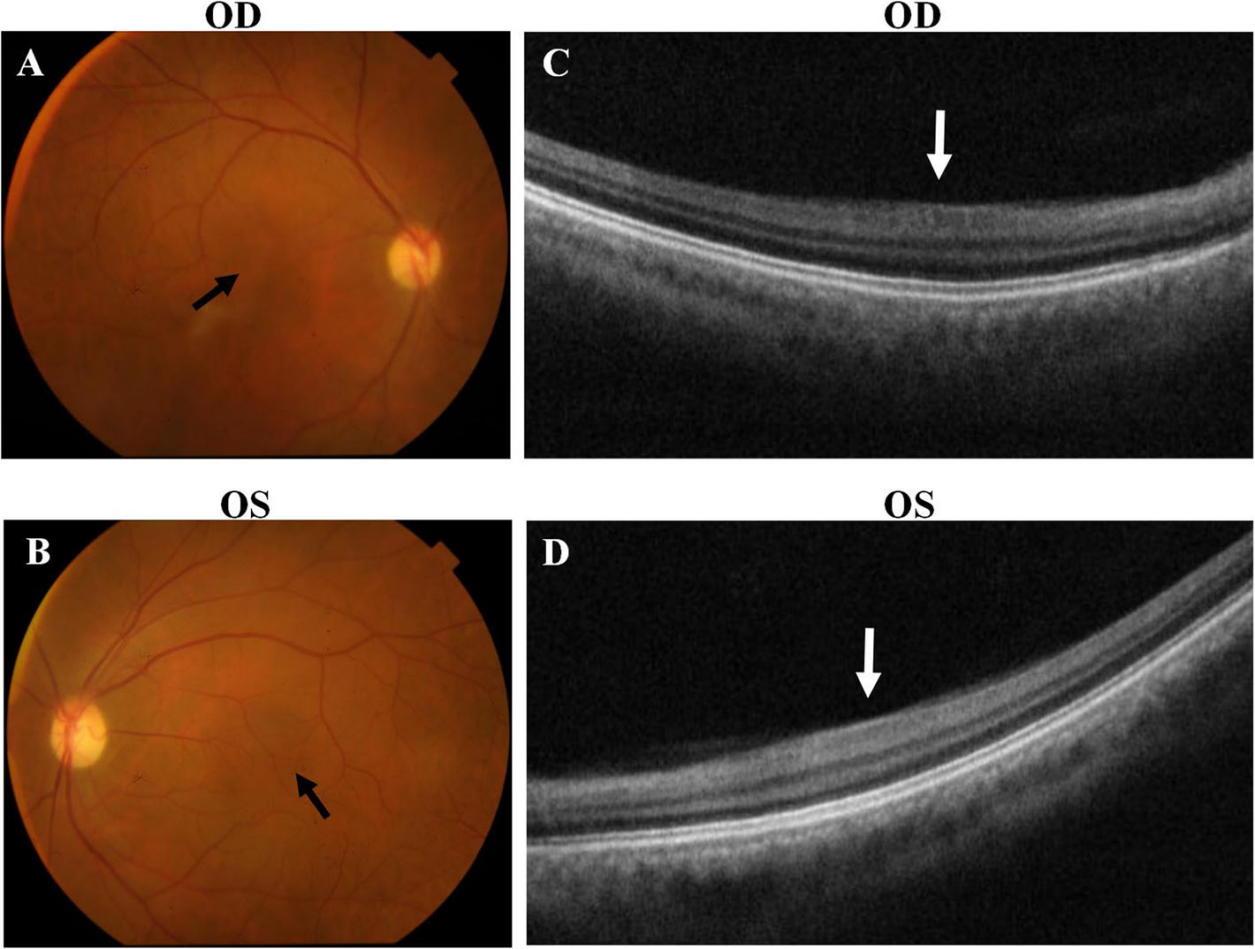
Fundus examination of the proband in the family with aniridia. **a** and **b** Fundus photography of proband. The black arrows showed the structure of macular area. **c** and **d** Macular OCT examination of proband.The white arrows indicated the structure of fovea of macula. OD stands for right eye, OS, left eye

**Fig. 4:**
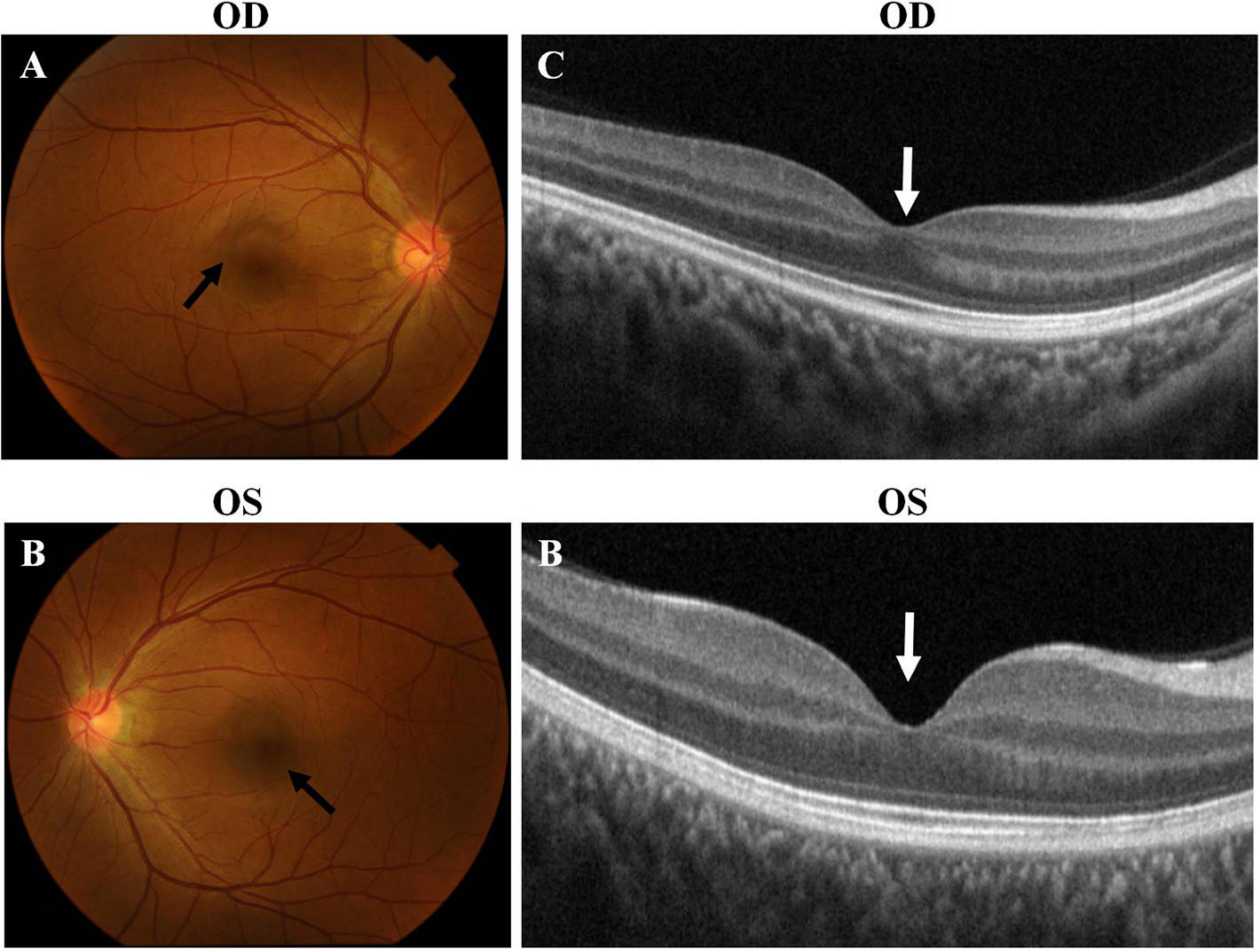
Fundus examination of the normal control. **a** and **b** Fundus photography of the normal control. The black arrows showed the structure of macular area. **c** and **d** Macular OCT examination of the normal control.The white arrows indicated the structure of fovea of macula

There were two affected individuals, the proband and his mother, in this two-generation Chinese family (Fig. 1a). The inheritance pattern of the pedigree was in accordance with autosomal dominant inheritance. The proband (II:4) was a 29-year-old male, he felt blurred vision and photophobia in both eyes since childhood. His BCVA were 0.2 in right eyes and 0.15 in left eyes, with corrections of -4.25 diopters (D) in the right eye and − 0.5 D in the left eye. Ophthalmic examination presented horizontal nystagmus, absence of almost whole iris, discrete posterior subcapsular cataract (Fig. 2a and b), and absence of macular central fovea (Fig. 3) in both eyes. The absence of iris was so severe that the equator of the lens and ciliary processes were exposed, which could be observed in anterior segment photography (Fig. 2b and f) and UBM examination (Fig. 2e). Anterior segment photograph demonstrated that the cornea of both eyes was transparent (Fig. 2a and b), but the central corneal thickness was 668µm in right eye and 664µm in left eye (Fig. 2 c and 2d), which were slightly thicker than normal. The structure of anterior chamber angle could be observed under gonioscopy, and the anterior chamber angle was open (Fig. 2f). The normal macula central fovea structure could not be found in fundus photograph and macular OCT (Figs. 3 and 4). Intraocular pressure of both eyes was normal. His mother had similar clinical symptoms and signs, moreover, her cataract became worse with age and she underwent phacoemulsification and intraocular lens implantation at 52 years old. There were no other systemic diseases except eye abnormalities in all the affects.

### Identification of causing mutations

After filtering the candidate variants of the proband in the databases, a nonsense mutation *PAX6* c.619A > T in exon 8 changing codon 207 AAG to the stopcodon TAG (p.K207X) was detected. The mutation was absent in either databases mentioned earlier or reported literatures, which led to Lysine at 207 position in linker region transforming to a premature termination codon, and finally resulted in *PAX6* underdosage (Fig. 1b). Then the novel mutation was validated by Sanger sequencing and detected among the family members, which demonstrated that the *PAX6* c.619A > T heterozygous mutation was cosegregated with the aniridia phenotype in this family (Fig. 1a). The affected individuals, the proband and his mother, carried the mutation, while the unaffected members did not. These results suggested that *PAX6* c.619A > T was a novel causative mutation for autosomal dominant congenital aniridia.

## Discussion

Aniridia associated with mutations in PAX6 was categorized into classic aniridia group, while that associated with mutations in genes other than *PAX6* was aniridia-like group [7, 8]. Classic aniridia referred to a pan-ocular disorder, including partial or near total absence of iris, cataract, aniridia-associated keratopathy (ARK), glaucoma, foveal hypoplasia, optic disk hypoplasia and nystagmus [14–16]. Iris hypoplasia was the most important feature of aniridia, which could range from complete absence of the iris, through enlargement and irregularity of the pupil mimicking a coloboma, to small slit-like defects in the anterior layer seen only on transillumination with a slit-lamp. The incidence of other features of classic aniridia was different, nystagmus was 76 %, cataract was 56 %, glaucoma was 64 % and visible keratopathy was 80 % [17]. In this study, we identified the pathogenic gene mutation in a Chinese aniridia family using an iris diseases panel including 37 targeted genes. The patients in this family presented nystagmus, ARK, absence of iris, cataracts and foveal hypoplasia. Then a novel *PAX6* nonsense mutation c.619A > T (p.K207*) was identified and it was co-separated from disease phenotype.

A high proportion of cases of aniridia was associated with mutations in *PAX6* frameshift mutations, splicing site mutations or nonsense mutations and these kinds of variations have been considered to produce premature truncation of the protein or nonsense transcripts, leading to haploinsufficiency. While few cases were caused by missense mutations [16]. Aniridia phenotype associated with *PAX6* haploinsufficiency almost present anterior segment and fundus abnormalities, while missense mutations in *PAX6* were mostly associated with dysplasia of skeleton and central nervous system [18]. The most common clinical manifestations associated with aniridia haploinsufficiency were iris anomalies, nystagmus and foveal hypoplasia, followed by cataracts, glaucoma and corneal opacity. In this Chinese family, the phenotype was similar between the two patients. they both felt photophobia from childhood and exhibited nystagmus, ARK, aniridia, cataract and foveal hypoplasia. The novel mutation *PAX6* c.619A > T (p.K207*) in this family induced premature termination codons (PTCs) into the *PAX6* open reading frame, and the mRNAs containing PTCs were degraded by the nonsense-mediated decay process, which resulted in a single-dose deficiency. The phenotype associated with the novel mutation was in line with classic aniridia related to *PAX6* haploinsufficiency [16].

It was reported that the severity of iris hypoplasia varied in different *PAX6* cases and lens abnormalities include various degrees and types of cataracts and lens ectopic [16]. The patients of this Chinese family presented near total absence of iris and lamellar posterior subcapsular lens opacification without obvious lens ectopia. Keratopathy was common in aniridia patients as the PAX6 gene is responsible for embryonic and postnatal development of the cornea. At the early stage of keratopathy, the central corneal thickness increased, the basal epithelium became turbid and the corneal sensitivity decreased. With the progression of the lesion, the cornea gradually became opacity from periphery area to apex [16]. In this family, the cornea of the proband remained transparent, but the central corneal thickness increased and the limbal vascular pannus indicated the presence of early ARK. Glaucoma in aniridia usually occurred in early adulthood, including infants and toddlers. It was caused by the irregular strands arising from the iris stroma attached to the angle wall. It was reported that a 24-year-old aniridia patient with *PAX6* c.607 C > T, p.Arg203* presented glaucoma, the location of the mutation was very close to that of *PAX6* c.619A > T (p.K207*), and these two gene locations were in the same domain [19]. However, the patients in this Chinese aniridia family were adults with normal intraocular pressure and no characteristic optic disc manifestations of glaucoma. They need follow-up observation of intraocular pressure to finally determine whether glaucoma will occur.

The human *PAX6* gene was cloned in 1991 and had been isolated from both vertebrates and invertebrates. It consisted of 14 exons and encodes a transcriptional regulator which had a paired-type DNA-binding domain. There were 2 distinct DNA-binding subdomains, the N-terminal subdomain (NTS) and the C-terminal subdomain (CTS), in the paired domain, which bind respective consensus DNA sequences to recognize target genes. The human *PAX6* gene produced 2 alternatively spliced isoforms that have the distinct structure of the paired domain [20]. There have been about 500 mutations reported in the human *PAX6* database [14] (https://www.ncbi.nlm.nih.gov/bioproject/PRJNA691696), since firstly identified as the genetic cause of aniridia in the small eye mouse [21]. While it was remarkable that the alterations in a conserved non-coding element within the “critical region” and other cis-regulatory elements also could cause aniridia [22]. *PAX6* whole-gene deletions and telomeric cis-regulatory elements deletions were also identified in some aniridia patients of negative for intragenic *PAX6* mutations. Consequently, it was suggested that *PAX6* whole-gene direct sequencing combined with and molecular methods of detecting copy number alterations (CNV) such as high-resolution comparative hybridization (HR-CGH) arrays, fluorescence in situ hybridization (FISH), and multiplex ligation-dependent probe amplification (MLPA) was important to improve detection rate for aniridia associated with *PAX6* variations, which could be more suitable for using in the aniridia cases of iris diseases panel screening negative [23]. It was thought greater locus heterogeneity might exist in both isolated and syndromic aniridia than was previously appreciated, therefore the improvement of detection method was helpful to improve the detection rate of pathogenic genes for aniridia. In this study, we just have screened protein-coding region of 37 genes associated iris diseases but ignored conserved non-coding element and CNV of *PAX6*, which should be improved in the future.

## Conclusions

In brief, a novel nonsense mutation in *PAX6* (c.619A > T p.K207*) was identified in a Chinese family with aniridia, which could cause *PAX6* haploinsufficiency. The phenotype associated with this mutation included aniridia, ARK, cataracts and foveal hypoplasia. The present study expanded the mutation spectrum of the *PAX6* gene which may be helpful in the genetic diagnosis of aniridia.

## Data Availability

All data generated and analyzed during this study were included in this manuscript.

## Abbreviations

WAGR: Wilms tumor, aniridia, genitourinary anomalies and mental retardation syndrome
NGS: Next-generation sequencing
IRB: Institutional review board
BCVA: Best correct visual acuity
UBM: Ultrasound biomicroscopy
OCT: optical coherence tomography
PAX6: Paired box gene 6
ELP4: Elongator acetyltransferase complex subunit 4
FOXD3: Forkhead box D3
PITX3: Paired-like homeodomain transcription factor 3
FOXE3: Forkhead box E3
PITX2: Paired-like homeodomain transcription factor 2
ADAMTS10: A disintegrin-like and metalloproteinase with thrombospondin type 1 motif, 10
FBN1: Fibrillin 1
LTBP2: Latent transforming growth factor-beta-binding protein 2
ADAMTS17: A disintegrin-like and metalloproteinase with thrombospondin type 1 motif, 17
MTHFR: Methylenetetrahydrofolate reductase
TYR: Tyrosinase
MITF: Microphthalmia-associated transcription factor
PAX3: Paired box gene 3
SNAI2: Snail family transcriptional repressor 2
SOX10: Sry-box 10
RBP4: Retinol-binding protein 4
CHD7: Chromodomain helicase dna-binding protein 7
TMEM67: Transmembrane protein 67
RPGRIP1L: Rpgrip1-like
CC2D2A: Coiled-coil and c2 domains-containing protein 2a
YAP1: Yes-associated protein 1, 65-kd
MAF: Maf bzip transcription factor
C12orf57: Chromosome 12 open reading frame 57
FOXC1: Forkhead box c1
B3GLCT: Beta-3-glucosyltransferase
NF1: Neurofibromin 1
GPR143: G protein-coupled receptor 143
OCA2: Oca2 melanosomal transmembrane protein
TYRP1: Tyrosinase-related protein 1
CRYGC: Crystallin, gamma-c
ADAMTSL4: Adamts-like 4
SALL2: Sal-like 2
IGBP1: Immunoglobulin-binding protein 1
CYP1B1: Cytochrome p450, subfamily i, polypeptide 1
MYOC: Myocilin
SALL1: Sal-like 1
SNPs: Single nucleotide polymorphism
PCR: Polymerase chain reaction
PTC: Premature termination codon
NTS: N-terminal subdomain
CTS: C-terminal subdomain
CNV: Copy number alterations
HR-CGH: High-resolution comparative hybridization
FISH: Fluorescence in situ hybridization
MLPA: Multiplex ligation-dependent probe amplification

## Data availability statement

The datasets generated during the current study are available in the https://www.ncbi.nlm.nih.gov/bioproject/PRJNA691696.
